# Preparation and Characterization of Boza Enriched with Nonfat Dry Milk and Its Impact on the Fermentation Process

**DOI:** 10.3390/gels10010022

**Published:** 2023-12-26

**Authors:** Ezgi Pulatsu, Sargun Malik, Mengshi Lin, Kiruba Krishnaswamy, Bongkosh Vardhanabhuti

**Affiliations:** 1 School of Nutrition Sciences, Faculty of Health Sciences, University of Ottawa, Ottawa, ON K1H 8M5, Canada; 2 RNAissance AG LLC, Biotechnology Research Overland Park, St. Louis, MO 63132, USA; sargunmalik@outlook.com; 3 Department of Food, Nutrition & Exercise Sciences, University of Missouri, Columbia, MO 65201, USA; linme@missouri.edu (M.L.); krishnaswamyk@missouri.edu (K.K.); vardhanabhutib@missouri.edu (B.V.); 4 Department of Biomedical, Biological and Chemical Engineering, University of Missouri, Columbia, MO 65211, USA

**Keywords:** colloids, fermentation, FTIR, gel, plant-based, rheology

## Abstract

Boza is an indigenous, traditional, low-alcohol and highly viscous beverage prepared by fermenting cereals. Its thick and gel-like consistency make it suitable for consumption via spoon. Although boza is a nutritious beverage, its protein content is very low (<2%). A new type of boza was developed by incorporating nonfat dry milk (NFDM) to elevate the protein content of the beverage. Different NFDM amounts (10 to 40% *w*/*v*) were added to determine the best concentration and fermentation time based on the refractive index and pH values at room temperature (0–48 h). The best sample was further characterized by rheological analyses and Fourier transform infrared (FTIR) spectroscopy. The sample with 10% NFDM was the best, as fermentation was successfully performed, and further addition of NFDM increased the initial pH. The refractive index and pH decreased from 21.9 ± 0.1 to 11.8 ± 0.1 and 5.77± 0.50 to 4.09 ± 0.35 during fermentation, respectively. The samples exhibited shear-thinning, solid-like behavior, and a gel-like structure. FTIR analysis by independent modeling of class analogy (SIMCA) demonstrated that unfermented slurry and the fermented product could be effectively differentiated. With the addition of 10% NFDM, the increase in the protein content of the boza medium became significant.

## 1. Introduction

Boza is a highly viscous fermented cereal beverage that originated in Central Anatolia. It is a healthy and nutritious beverage due to its lactic acid content [1]. It also has the ability to improve digestion and intestinal flora [1]. This indigenous and traditional beverage is made from different raw materials, depending on the region or country, and it is considered as a local product [2]. Boza has been associated with many countries, including Albania, Bulgaria, Bosnia and Herzegovina, Kazakhstan, Kyrgyzstan, Macedonia, Montenegro, Romania, Serbia, and Turkey [3]. Similar millet-based fermented beverages also exist in different countries [1]. Even though modern production relies on controlled fermentation with starter cultures, most of the time such traditional foods are the products of uncontrolled and spontaneous fermentation, as has been practiced for centuries.

Boza is described as a colloidal suspension from the food science perspective. Its color ranges from light to dark beige. Its taste ranges from sweet to slightly sharp to slightly sour. It is made from wheat, rye, millet, maize, and other cereals, mixed with sugar or saccharine [4]. These types of fermented beverages can be manufactured through a set of processes such as steeping, milling, slurrying, sieving, fermentation, sedimentation, and cooking [1]. Fundamentally, the cereal is cooked for several hours and then strained to remove the solids. Then, sugar is introduced, together with a starter culture, to initiate the fermentation at 30 °C for 24 h. After the subsequent cooling process, the product can be stored for 3–5 days for consumption [5]. Owing to its lactic acid, fat, protein, carbohydrate, fiber, and vitamin contents, boza can be recognized as a valuable fermented food contributing to human nutrition. Furthermore, its processing results in improved taste and quality, together with digestible compounds, as mentioned by [6]. Boza contains approximately 0.50–1.61% protein, 12.3% carbohydrate, and 75–85% moisture. The pH range of boza samples ranges from 3.16 and 4.02, and the average alcohol content of Turkish boza samples was found to range from 0.03 to 0.39% (*w*/*v*) [7].

From a microbiological point of view, *Lactobacillus*, *Lactococcus*, *Leuconostoc*, *Pediococcus*, *Enterococcus*, *Oenococcus*, and *Weissella* are the dominant genera isolated from boza samples known to be rich in lactic acid bacteria (LAB). On the other hand, some molds and yeasts have been isolated from traditionally fermented boza, where the previous batch was used as a starter culture [8]. *Candida* and *Saccharomyces* are the two major isolated yeast genera from boza. Bulgarian boza has been studied for microflora identification in which yeasts and LAB have been identified in an average LAB/yeast ratio of 2.4 [8,9].

The pourable and semi-solid nature of boza can be characterized by rheological studies, including steady-shear and oscillatory measurements. As stated by Owens et al. [10], the study of the flow behavior of food ingredients and products has potential widespread impact on the economies of production, public health, and individual diets. Rheological study is particularly insightful for understanding and adjusting properties such as the texture, drinkability, and mouthfeel of food products with multiple ingredients and time-dependent properties [10]. The thick and gel-like consistency of this drink, with distinct characteristics, has been identified in several studies using steady-shear and oscillatory measurements [11,12,13,14]. Viscosity, storage modulus, and loss modulus are the most-used rheological parameters in these studies. Model-fitting parameters such as the consistency coefficient and the flow behavior index are also discussed. For instance, random boza samples from local markets in Turkey exhibited gel-like behavior, together with shear-thinning behavior, as described by [11].

Plant-based beverages are generally considered functional and sustainable carriers of bioactive compounds with potential health benefits [15]. However, compared to dairy products, they have poor emulsion conditions, a light and bitter taste, and insufficient nutritional components [15]. One way to alter their properties is through fermentation, in which direct metabolites and secondary metabolites are produced under the specific survival conditions of microorganisms that are beneficial to the taste, flavor, and functional properties of the beverages [15].

The increasing demand for dairy and non-dairy fermented beverages, along with the potential health implications, led to increased research and publications in this area, as pointed out by Marrero et al. [16]. However, except for the studies of Arslan-Tontul and Erbas [17] and Celik et al. [13], no other study exists about the protein enrichment of boza, even though the protein content has been reported to be low. The addition of gluten, zein, and chickpea flour improved the protein content, while volatile compounds formed due to the activity of *Lactobacillus acidophilus*, *Bifidobacterium bifidum*, and *Saccharomyces boulardii* during the co-culture fermentation of the cereal medium [17]. Owing to the addition of gluten, the protein content of the boza medium increased by up to 271% [17]. Celik et al. [13] investigated the substitution of cereal flour (maize, rice, and wheat) with yellow chickpea flour, which yielded the highest protein content (8.46%) among the samples.

Proteins are among the most important components of diet. They are a good source of energy and overall nutrition that help in maximizing the performance of the human body [18]. Since one-third of the world’s population suffers from protein deficiency [17], there is a significant need to fortify food products with diverse protein sources, including plant-based and animal proteins. Animal proteins are considered the highest quality proteins with an adequate amount of essential amino acid [19]. Along with the necessary nutrition, they are also known for their functional benefits, including solubility and heat stability, as well as for their foaming and gelling properties [20]. One of the popular animal-sourced proteins is nonfat dry milk (NFDM). NFDM has a mild taste due to the lack of fat and, therefore, it is generally used to enhance the nutritional profile of non-dairy products. Therefore, the use of NFDM in boza is viable to boost the protein content of the traditional product.

Owing to their ready-to-eat forms, fermented ethnic foods and industrial probiotic foods fulfill the current requirement of delivery vehicles of bioactive compounds and nutrients at convenience [3,21,22]. Behera and Panda [21] point out that improvement of the nutritional value of ethnic foods will be the priority area in the near future. Hence, the objective of this study is to create a beverage inspired by a traditional recipe by incorporating a dairy ingredient, NFDM, that will help improve its nutritional profile, especially its protein content. The research focuses on studying the quality attributes of nonfat dry milk with boza by investigating pH and the refractive index as well as rheological and Fourier transform infrared (FTIR) spectroscopy characteristics.

## 2. Results and Discussion

### 2.1. The pH and Refractive Index Results

**Figure 1:**
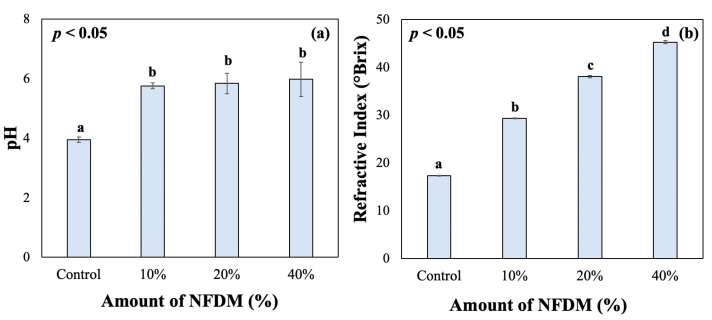
The effect of NFDM content on the initial pH (**a**) and RI values (**b**).

The change in the initial pH values of all samples is given in Figure 1a. The addition of NFDM increased the initial pH values significantly (*p* < 0.05); however, further addition of NFDM did not affect the pH values significantly (*p* > 0.05). In other words, samples with 10 to 40% NFDM had similar pH values (*p* > 0.05) (Figure 1a). For boza and other fermented beverages, measuring pH helps determine the fermentation duration and estimate the growth of LAB. In this study, pH values were used to designate the initial pH values and compare the samples with varying NFDM content.

In addition to initial pH, initial RI values of the samples were determined, as shown in Figure 1b. It was found that initial brix values of the 10%, 20% and 40% (*w*/*v*) samples were 29.4, 38.1 and 45.3 as compared to 17.4 of the control sample (Figure 1b). The increase in RI was in line with the findings that the RI increases with an increase in total solids in the formulation [23].

The initial pH values were 5.77 for 10% (*w*/*v*), 5.84 for 20% (*w*/*v*) NFDM, and 5.98 for 40% (*w*/*v*) NFDM as compared to the control sample which was at 3.95. Even though the initial pH is not significantly different among the NFDM-containing samples, as given in Figure 1a, it becomes harder to accommodate the pH drop with the same amount of starter culture within the duration of fermentation. This might be because of the increased viscosity of the samples (results were not shown) when more NFDM was incorporated, which affected the microbial growth and their diffusion within the sample. The NFDM addition at 20–40% yielded very thick slurries (visual observation), which were significantly different from the consistency of the control sample (results are not shown). Therefore, based on the pH, RI values and appearance of the sample in terms of foaming and bubble formation, 10% (*w*/*v*) was selected for the formulation of the beverage. Moreover, NFDM or protein enriched beverages in the literature, is generally at 10% (*w*/*w* or *w*/*v*), which is in line with the present study [17,24].

In general, the pH of NFDM is in the range of 6.5–6.7 and the titratable acidity varies from 0.1 to 0.15%. In a study conducted by [25], the relationship between titratable acidity and pH was observed in a fermentation process of nonfat milk. It was observed that during the fermentation, the acidity increases for every component of the powder that is reconstituted [25,26]. Lactic acid bacteria grow readily in the food substrates and lower the pH rapidly to a point where competing organisms are no longer able to grow. They help in the preservation with comparatively lower energy requirements and consequently lower cost. The preservation occurs with nicotinamide adenine dinucleotide (NADH) oxidation that further reacts with oxygen [27]. During fermentation, free fatty acids are formed due to the lipolysis of fat in the bulgur grain, which contributes to decreased pH. The results were similar to a study conducted wherein cereal grains such as sorghum, maize and other millets were fermented using LAB and pH was found to be a critical indicator for estimating the LAB growth [28].

**Figure 2:**
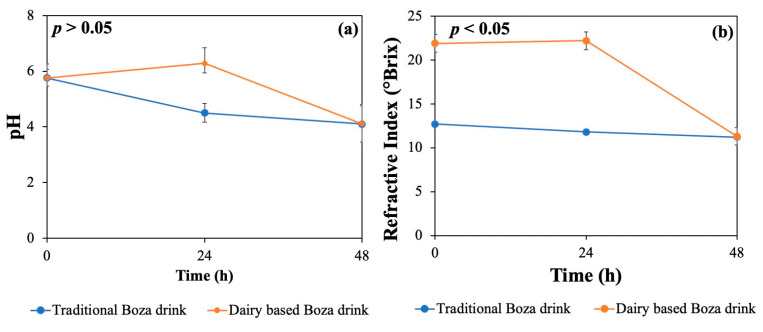
Change in pH values of traditional and 10% (*w*/*v*) NFDM dairy-based boza during fermentation (**a**); change in RI values of traditional and 10% (*w*/*v*) NFDM dairy-based boza during fermentation (**b**).

During fermentation, both traditional boza and dairy-based boza beverages exhibited a decrease in pH, with the traditional beverage sample showing a rapid drop compared to dairy-based sample. After 24 h, the pH values were 4.49 and 6.28 for traditional and dairy-based boza. There was no significant change in pH for the dairy-based sample after 24 h. After 48 h, the dairy-based sample went through a similar pH drop wherein both the samples had a pH value of 4.09, as shown in Figure 2a, indicating a slow fermentation in the dairy-based sample. The RI values decreased in both traditional boza and dairy-based boza during fermentation Figure 2b. The initial RI for traditional and dairy-based samples were noted as 12.7 and 21.9° Brix, respectively. After 24 h fermentation, a drop in RI was observed in the traditional boza sample, but that of the dairy-based sample remained similar to its initial value. As depicted in Figure 2b, after 48 h fermentation, a drastic change was observed in the dairy-based sample, wherein it reached 11.8° Brix, which was similar to the traditional sample. The RI values obtained in the experiment were indicative of the composition and soluble dry matter content of the sample. During fermentation, microorganisms use sugars and convert them to ethyl alcohol and carbon dioxide. Hence, the RI could be used to represent the progress of the fermentation process. A similar method was used for analyzing the fermentation process in a rice-based fermented product called *Tape Ketan* in a study conducted by [29]. RI was used for alcohol measurement, and the study concluded that as the fermentation progressed, there was an increase in alcohol content and a decrease in dissolved sugar, justifying the reduction in RI.

RI also suggested the amount of NFDM that could be added in the traditional beverage to obtain a dairy-based beverage. Hence, the sample containing 10% (*w*/*v*) NFDM was finalized for further experimentation. Given that NFDM contains 34% protein, the protein content could be increased significantly.

### 2.2. Rheological Properties of Dairy-Based Slurry and Boza

**Figure 3:**
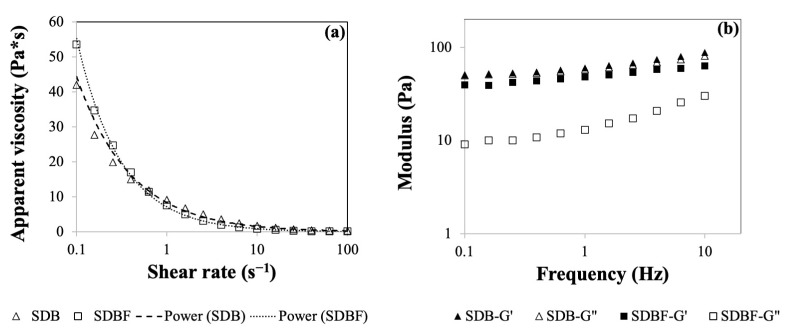
Apparent viscosity vs. shear rate (**a**) and frequency sweep results (**b**) of dairy-based boza and unfermented slurry.

As shown in Figure 3a, the apparent viscosity of fermented (SDB-F) boza and unfermented dairy-based slurry (SDB) showed shear-thinning behavior evident by the reduction in the apparent viscosity values over the increased shear rate. The related model parameters were tabulated in Table 1. The correlation coefficients for the model fitting were greater than 0.92, which ensures a successful data fitting. Both samples exhibited similar behavior in the given range of shear rates (*p* ≤ 0.05). Fermentation did not affect the flow behavior of the sample significantly (*p* ≤ 0.05). Also, n values were less than 1, which proved the shear-thinning behavior of the samples (Table 1). Hence, both consistency and the flow behavior index were not affected by fermentation, which might be related to low alcohol content and CO_2_ levels reached during fermentation.

In the literature, the rheological properties of boza have been studied, and similar findings have been reported [12]. However, there is no report on the characterization of the slurry before fermentation. The current study indicated no significant change in the flow properties of the slurry and fermented product.

After characterization of the viscosity of the samples, dynamic rheological testing was performed, as shown in Figure 3b. The storage and loss modulus of the samples were plotted against the frequency range. The results demonstrated that for the given frequency range, solid-like behavior dominated the overall behavior since $G^{\prime }$ > $G^{\prime \prime }$, for both samples (Figure 3b). It can be inferred that the samples are gel-like structures with limited frequency dependency in the given frequency range. The viscoelastic nature of the samples was successfully captured via dynamic rheological measurements aligning with the literature reports [11]. Additionally, model fitting enabled sample comparison to designate if there is a significant difference using statistical analysis. No significant difference was found between the samples at the 0.05 significance level, which aligned with the viscometry results. Both fermented product and unfermented slurry exhibited similar rheological behavior.

### 2.3. FTIR Spectroscopy

**Figure 4:**
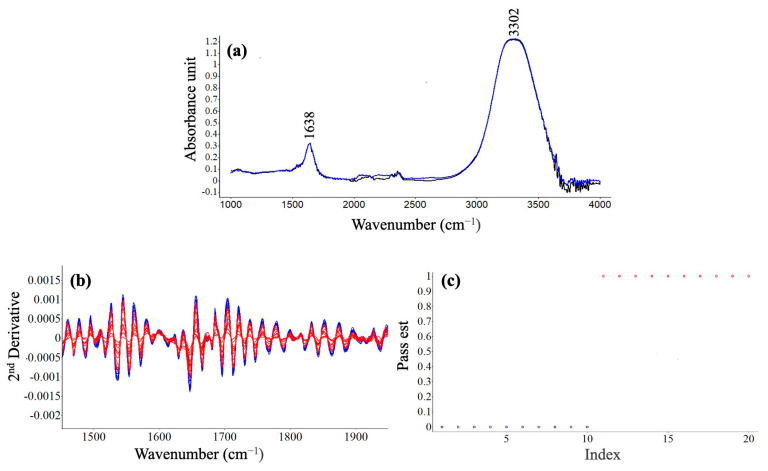
FTIR absorbance spectra of SDB-F (blue) and SDB (black) representing the average of ten sample measurements (**a**), the second derivative of all spectra: red represents SDB, and blue represents SDB-F (**b**), and SIMCA result: blue (10 replicates of SDB-F sample spectra) and blue (10 replicates of SDB sample spectra) (**c**).

Figure 4 presents FTIR spectra and related SIMCA test results to differentiate the fermented sample (SDB-F) from the unfermented sample (SDB). Two major peaks, 1638 and 3302 cm^−1^, observed within the scanned range (Figure 4a). The first peak can be correlated with the amide bands corresponding to proteins, or more specifically C=O stretching vibrations of amides of proteins [30]. Kher et al. [31] reported that the bands in the amide region (1700–1600 cm^−1^) have previously been assigned to the α-helix and that at approximately 1630 cm^−1^ to the β-sheet by several authors [32]. The 1638 cm^−1^ can be attributable to β-sheet structures resulting from NFDM. Hence, the findings were in good agreement with the literature [31]. The latter peak can be associated with O–H stretch of water.

Several alcoholic compounds can be formed during cereal fermentation, n-propanol, isobutanol, amyl alcohol, isoamyl alcohol, 2,3-butandieol, and β-phenylethyl alcohol [33]. Three specific spectral regions of 3005–2960 cm^−1^, 1200–950 cm^−1^ and 900–850 cm^−1^ were reported to be ethanol-related peaks [34].

The second derivative spectra were analyzed to elucidate small spectral differences among the samples, as shown in Figure 4b. The intensities from the fermented product were stronger than the unfermented slurry in the scanning range (Figure 4b). Furthermore, SIMCA was applied to the spectra using a pass test, where the pass value will be true (1) or false (0) according to the base spectra selected. The results indicated that SDB-F and SDB samples are different from each other, as expected, due to several biochemical changes due to fermentation. All ten replicates of each group were successfully discriminated against via SIMCA.

This study demonstrates that FTIR spectroscopy, in conjunction with SIMCA, can consistently differentiate samples of fermented dairy-based boza and unfermented dairy-based slurry. However, a more detailed analysis should be performed to designate biochemical changes during fermentation which will be performed in future studies.

## 3. Conclusions

The incorporation of NFDM was successfully achieved to obtain a dairy-based boza, leading to increase in protein content given that NFDM contains 34% protein. However, the addition of NFDM resulted in an increased initial pH of the slurry that prolonged the fermentation time to 48 h. Both pH and RI values decreased during the course of fermentation, as expected. Based on the physicochemical analyses, the sample with 10% NFDM was designated as the best sample. According to the results of rheological characterization, the samples are shear-thinning, solid-like behavior, and gel-like structures. FTIR analysis, together with chemometrics methods, can differentiate the unfermented slurry from the fermented product up to 100% of the time. Thus, this method has great potential to study cereal-based fermented beverages.

This study is a step toward the research efforts in the development of enriched food products with diverse ingredients. Millet-based agricultural products are abundant, and their utilization in various food products is nutritionally and economically promising. New consumer trends are in the direction of ethnic/traditional foods and beverages; therefore, dairy-based boza can be a good alternative for finding healthy, natural, and nutritious ethno-food. This preliminary study is unique in terms of evaluating the suitability of NFDM addition into plant-based boza beverage. Further research is necessary to analyze the biochemistry profile as well as the protein digestibility of this unique fermented dairy-based beverage. Also, more characterization techniques should be employed to discuss the properties of the product further, including sensory properties.

## 4. Materials and Methods

### 4.1. Materials

Sugar and nonfat dry milk (instant dry milk, Great Value) were purchased from the local market (Columbia, MO, USA). Bulgur (Duru, fine bulgur, 11 g protein/100 g) and a commercial boza were supplied from Turkey (Omur Boza).

### 4.2. Boza Preparation

With an extensive search in the literature and preliminary analyses, the boza recipe was established. Briefly, 1 cup of bulgur (160 ± 10 g) was soaked in water overnight. Then, the soaked bulgur was boiled in 1.5 L of distilled water for 1.5 h. Half of the bulgur grains were removed to obtain better consistency, and the remaining mixture was passed through the sieve (1 mm), which was designated by preliminary experiments. The obtained solution was blended with a kitchen hand blender for 1 min and cooled down to 40 °C in the refrigerator. To the blended mixture, 20 g of boza as a starter culture, 37.5 g of sugar and 100 mL of distilled water were added. The whole mixture was then left for fermentation at room temperature for 48 h.

### 4.3. Dairy-Based Boza Preparation

**Figure 5:**
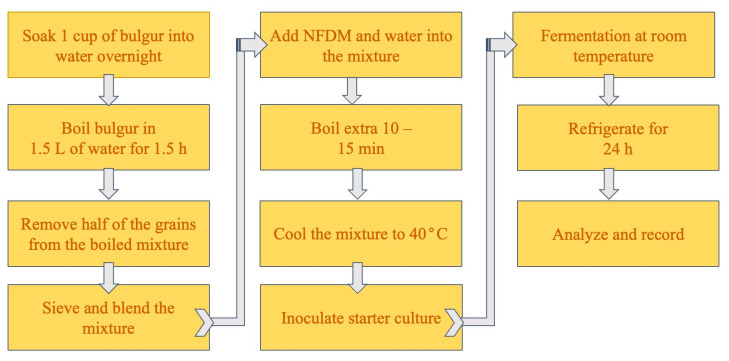
Flow diagram of dairy-based boza production.

Similar to traditional boza preparation, dairy-based boza was obtained, as shown in Figure 5. Additionally, 50 g of NFDM was mixed with 100 mL water to obtain the dairy-based slurry. The mixture was then boiled for 10–15 min and cooled down to 40 °C. Sugar (37.5 g) was then added together with 20 g of boza starter culture. The mixture was then left for fermentation at room temperature for 48 h. Upon completion of fermentation, samples were placed in the refrigerator for 24 h prior (which is a common practice before the consumption of the fermented beverage) to rheological and spectral analyses.

### 4.4. The pH and Refractive Index Measurements

The pH of the samples was measured using a digital pH meter (Mettler Toledo™, Columbus, OH, USA) with a pH electrode (In Lab^®^ Expert Pro-ISM, Columbus, OH, USA). The refractive index (RI) was measured using digital refractometer (WM-7 ATAGO™, Bellevue, WA, USA), which provides information regarding the water-soluble dry matter content. Traditional boza was used as a reference sample to determine the fermentation time for dairy-based boza, as this is the first study exploring dairy-based boza. The pH and RI of traditional and dairy-based boza were measured at 0, 24 and 48 h after fermentation. Then, the fermentation time was designated when the Brix° and pH were the same for both traditional and dairy-based boza samples.

### 4.5. Rheological Measurements

Rheological characterization of selected samples was carried out using a Kinexus Pro rheometer (NETZSCH Instruments North America, LLC, Burlington, MA, USA) operated with a parallel plate geometry of 50 mm diameter and a 2 mm gap. Fermented dairy-based boza (FDB) and unfermented dairy-based slurry (DB) (before the addition of starter culture) were selected for the analysis after successful trials. The samples were taken out to room temperature (25 ± 1 °C) for one hour before the rheological analysis. Viscosity measurements were carried out in the shear rate range of 0.1–100 s^−1^. All measurements were performed in triplicate. The power-law model fitting was carried out to describe the flow behavior of the samples (Equation (1)).

(1) $$\sigma =\eta {\dot{\gamma }}^{n}$$

where *σ* represents stress (Pa), *ƞ* is the viscosity, $\dot{\gamma }$ represents the shear rate, and n is the power of the rate. Then, the equation was converted to Equation (2) to represent the K (Pa ∗ s^n^) and n (dimensionless) parameters which are the consistency coefficient and the flow behavior index, respectively [35].

(2) $$\eta ={\left(K\dot{\gamma }\right)}^{n-1}$$

For the dynamic rheological properties of the products, amplitude sweep tests (at 1 Hz in the range of 0.01–100% strain) were run to determine the linear viscoelastic region (LVER) prior to frequency sweep measurements. Frequency tests were performed at 0.1% shear strain in the frequency range of 1–10 Hz, where storage ($G^{\prime }$) and loss ($G^{\prime \prime }$) modulus were recorded to describe the rheological behavior of the samples.

$G^{\prime }$ and $G^{\prime \prime }$ values obtained from the frequency tests were fitted in the model as described in the following equations.

(3) $$\log G^{\prime }=\log a^{\prime }+b^{\prime }\log\omega$$

(4) $$\log G^{\prime \prime }=\log a^{\prime \prime }+b^{\prime \prime }\log\omega$$

where *ω* rad/s is the angular frequency and $a^{\prime }$, $a^{\prime \prime }$, $b^{\prime }$, and $b^{\prime \prime }$ are the model parameters. The frequency dependence of the samples was quantified via these model parameters. Among them, $a^{\prime }$ and $a^{\prime \prime }$ are the flow coefficients, $b^{\prime }$ and $b^{\prime \prime }$ indicate the frequency sensitivity of the samples [36].

### 4.6. FTIR Analysis of Dairy-Based Boza

FTIR spectra of selected samples, SDB-F and SDB, were collected by an FTIR spectrometer (Nicolet 380; Thermo Fischer Scientific, Waltham, MA, USA), in the region of (4000–400 cm^−1^) with attenuated total reflection mode. FTIR spectra (n = 10) of each sample were collected where the resolution was set as 2 cm^−1^ with an average of 32 scans for each spectrum to be analyzed using Delight version 3.2.1 (D-Squared Development, LaGrande, OR, USA) software. First, data processing was performed, where spectral data were merged and smoothed with a Gaussian function (over 12 cm^−1^). Then, a second derivative transformation with a 12 cm^−1^ gap was performed [30]. SIMCA classification was performed on all the spectral data collected.

### 4.7. Data Analysis and Statistics

Experimental data were given as the mean ± standard deviation for pH, RI and rheological parameters. The significant differences among the data points were evaluated based on one way analysis of variance (ANOVA) and Tukey’s test (Minitab, Version 16.2.0.0, Minitab Inc., Coventry, UK) with a *p*-value of ≤0.05. For the spectral analysis, SIMCA was used, which is a classification method based on principal component analysis (PCA), a common multivariate statistical technique.

## Figures and Tables

**Table 1:** The results of model fitting for viscometry and oscillation. (Means sharing the same letters (a) are statistically the same (*p* ≥ 0.05). Each parameter was analyzed separately).

Sample	Model Fitting Parameters for Viscometry and Oscillation		
	Model fitting parameters for viscometry		
	K	n	R^2^
SDB-F	7.10 ± 0.43 ^a^	0.11 ± 0.05 ^a^	0.92
SDB	8.36 ± 0.38 ^a^	0.28 ± 0.01 ^a^	0.98
	Model fitting parameters for oscillation		
	a’	b’	R^2^
SDB-F	41.16 ± 17.72 ^a^	1.39 ± 0.17 ^a^	0.96
SDB	47.04 ± 17.52 ^a^	1.35 ± 0.04 ^a^	0.93
	Model fitting parameters for oscillation		
	a”	b”	R^2^
SDB-F	39.97 ± 17.57 ^a^	1.35 ± 0.16 ^a^	0.94
SDB	47.04 ± 17.52 ^a^	1.30 ± 0.03 ^a^	0.93

## Data Availability

The data presented in this study is already available in the manuscript. Additional information can be provided by the corresponding author upon request.
